# Ischemic stroke on SARS‐CoV2 vasculitis in a healthy young girl

**DOI:** 10.1002/hsr2.1046

**Published:** 2023-01-23

**Authors:** Hortense Petat, Adnan Hassani, Ivana Dabaj, Lucile Tzaroukian, Barbara Goujard, Isabelle Michelet, Rebecca More

**Affiliations:** ^1^ U1311 DYNAMICURE Université de Rouen Normandie ‐ IRIB ‐UFR Santé‐ 22 Boulevard Gambetta‐ CS 76183‐ 76183 Rouen France; ^2^ Département de Pédiatrie Médicale Centre Hospitalier Universitaire de Rouen, EA2656 Université de Normandie, UNIRouen Rouen France; ^3^ Département de Radiologie Pédiatrique Centre Hospitalier Universitaire de Rouen Rouen France; ^4^ Département de Pédiatrie Néonatale Neurologie Pédiatrique, Centre Hospitalier Universitaire de Rouen Rouen France; ^5^ Département de Pédiatrie Néonatale Réanimation Pédiatrique, Centre Hospitalier Universitaire de Rouen Rouen France

**Keywords:** children stroke, COVID‐19, infectious vasculitis

## Abstract

**Background and Aims:**

In France, we noted the fifth wave of SARS‐CoV2 pandemic, characterized by presence of Omicron variant. This variant is very contagious, but less often aggressive, especially in pediatric population.

**Methods:**

We report a case of a 10‐year‐old girl, previously healthy, not yet vaccinated for SARS‐CoV2, presented to our emergency department for left hemiparesis associated with headache and vomiting, without any signs of respiratory tract infection.

**Results:**

Cerebral CT and MRI showed an ischemic stroke of right sylvian artery. Magnetic resonance angiography performed upon resurgence of new symptoms was in favor of vasculitis on the right internal carotid and right sylvian artery. PCR SARS‐CoV2 was positive for Omicron variant. She fully recovered after few days and was treated with acetylsalicylic acid and intravenous corticosteroids.

**Conclusion:**

We report this case to raise awareness on the possible complications related to SARS‐CoV2 infection and we highly recommend vaccination in this age group.

**Abbreviations**
ED: emergency department
FCA: focal cerebral arteriopathy
IgG: immunoglobulins G
MISC: multisystem inflammatory syndrome in children
NIHSS: National Institute of Health Stroke Score
PIMS: pediatric inflammatory multisystem syndrome
SARS‐CoV2: severe acute respiratory syndrome coronavirus 2

## 1 INTRODUCTION

For the last 2 years, the world has suffered a huge pandemic caused by severe acute respiratory syndrome coronavirus 2 (SARS‐CoV2). About 6 billion people died, and more than 350 billion people were affected by this virus. The succession of “viral waves” is characterized by the resurgence of different variants, due to permanent mutations and adaptation of the virus. In pediatric population, SARS‐CoV2 is rarely a symptomatic infection, and complications are quite limited.^1^ Specific presentations like multisystem inflammatory syndrome in children (MISC) are described, and target's population is defined.^2^ The underlying mechanism of this clinical presentation is not clearly understood yet, but it seems related to uncontrolled inflammatory response and cytokine storm after a SARS‐CoV2 infection.^3^, ^4^ Neurologic complications caused by SARS‐CoV2 are described.^5^, ^6^ (intracranial hemorrhages, acute strokes, large‐vessel arterial occlusions, ADEM, Guillain‐Barré syndrome, myelitis, necrotizing‐hemorrhagic encephalitis in adults, cytotoxic lesion of the corpus callosum).^7^ Large‐vessel cerebral arteriopathy is the most common cause of arterial ischemic stroke in previously healthy child.^8^ Focal cerebral arteriopathy (FCA) was defined recently by Wintermark et al. as unifocal and unilateral stenosis or irregularity of the distal internal carotid artery and/or its proximal branches. It can be inflammatory with a marked concentric vessel wall enhancement, or traumatic, like a dissection. Inflammatory arteritis could be caused by viruses. Respiratory viruses like varicella are known to possibly cause focal vasculitis in children. Only few cases of FCA during a SARS‐CoV2 infection are published.^6^ Here, we report the case of ischemic stroke secondary to a FCA during an asymptomatic SARS‐CoV2 infection in a young healthy girl.

## 2 CASE DESCRIPTION

A previously healthy 10‐years‐old girl came to emergency department (ED) at Rouen university hospital, in France, for syncope. She was not vaccinated against SARS‐CoV2. She fell in the stairs after a collapse, as she was alone at home. At the ED, she had intense headache, intense vomiting, and a left hemiparesis with moderate motor deficit. National Institute of Health Stroke Score (NIHSS) was 2. Because of these symptoms, she was hospitalized in the intensive care unit. After 48 h of monitoring, NIHSS was 0, and she was transferred to the conventional unit. At Day 4, she suffered from resurgence of headache as well as transient symptoms of left hemiparesis (tingling, limbs paresthesia, loss of muscular strength, and central facial paralysis). Every symptom, except central facial paralysis, lasted about 10–35 min. Central facial paralysis disappeared completely at Day 7. No sequalae was observed at Day 7 except mild asthenia. The patient never presented fever or any symptoms of acute respiratory tract infection before or during the hospitalization.

## 3 IMAGING

Brain CT, MRI (realized 7 h after the beginning of the symptoms) in addition to cerebral and cervical MRA were performed and showed an ischemic stroke of deep territory of right Sylvian artery with almost complete interruption of flow within the internal carotid artery (Figure 1.1). MRI T1 weighted sequence showed a thickening of the wall of the internal carotid artery in its intracranial portion associated with as significate increase of blood flow at this level (Figure 1.2). No image of thrombus was seen. MRI with angiography and contrast media injection was performed on Day 4 because of the apparition of new symptoms. It showed an extension of ischemia in the right deep sylvian territory, and signs of vasculitis on intracranial portion of the right internal carotid and proximal portion of right Sylvian artery (Figure 2.1). Note the partial recanalization of the right internal carotid on this exam (Figure 2.2).

**Figure 1:**
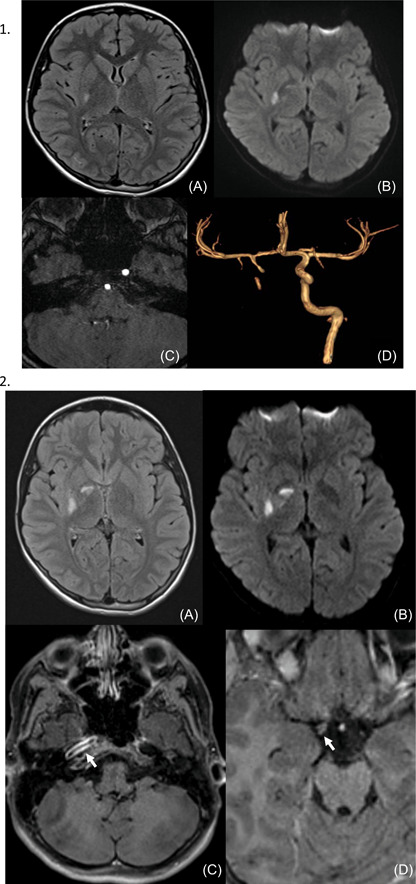
(1) Flair (A) and diffusion (B) sequences showing recent ischemic capsulo‐lenticular involvement and 3DTOF sequence in axial plane (C) and VR reconstruction (D), showing an almost complete absence of internal carotid flow throughout its height. (2) Flair (A) and diffusion (B) sequences showing an extension of ischemia in deep Sylvian territory and enhanced FatSat T1 sequence (C, D), showing enhancement of a circumferential parietal thickening of the right internal carotid artery, extending on the proximal portion of the right Sylvian artery

**Figure 2:**
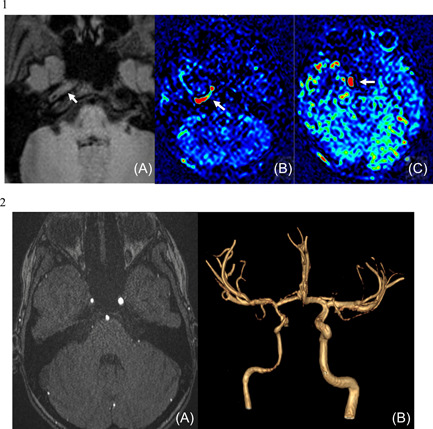
(1) T1 FatSat sequence showing circumferential thickening of the intracranial portion of the internal carotid artery and ASL sequence showing increased blood flow in the wall of the internal carotid artery in the intrapetrous portion (B) and in the intracavernous portion (C). (2) 3DTOF axial slice (A) and VR reconstruction (B) showing recanalization of the right internal carotid artery, but with decreased caliper relative to the controlateral artery

Trans‐thoracic echography did not show valvular defect or coronary artery dilatation. ECG was normal.

## 4 DIAGNOSIS ASSESSMENT

Blood count and blood electrolytes were normal. There was no inflammatory syndrome. d‐dimers were normal. There was no coagulation abnormality. Diagnosis of COVID‐19 was made according to the presence of SARS‐CoV2 viral nucleic acid in a naso‐pharyngeal swab using 2019 novel coronavirus real‐time reverse‐transcriptase polymerase chain reaction assay. Virus sequencing found Omicron variant. SARS‐CoV2 serology was negative for Immunoglobulins G (IgG). Other viral serologies (HIV, Epstein Barr Virus, Human Herpes Virus 6, Parvovirus B19, Cytomegalovirus, type B Hepatitis Virus) were negative. Research for autoimmune pathologies leading to vasculitis were negative. This ischemic stroke was so classified “unknown” based on the TOAST criteria.^9^

Acetylsalicylic acid was prescribed immediately at the ED (5 mg/kg/day). No anticoagulation treatment was initiated. Intravenous corticosteroids (30 mg per kilogram per day, 1000 mg per day) were administered at Days 7, 8, and 9 with scope monitoring. Then oral corticosteroids were administered at Days 10, 11, and 12 at the dose of 2 mg per kilogram per day, and at Days 13, 14, and 15 at the dose of 1 mg per kilogram per day. Potassic and calcic supplementations were given daily during corticosteroid treatment. No adverse events were noted except nocturnal agitation.

## 5 DISCUSSION

Here we report the case of focal cerebral arteriopathy in a young girl during infection by SARS‐CoV2 with Omicron variant, revealed by an ischemic stroke, in January 2022. Initial management was only acetylsalicylic acid but new symptoms on Day 4 convinced us to treat the patient with high doses of intravenous corticosteroids. A total regression of the symptoms was noted at Day 7.

Neurologic manifestations of SARS‐CoV2 infection are rare. A systematic review published on December 2021^10^ identified 41 cases of neurologic manifestations during 1 year (December 2019 and December 2020). Stroke was the most common neurologic diagnosis (pooled prevalence 2%), but it was described only in adults patients in this study. In adults, SARS‐CoV2 is known to have a 7.6 times increase (95% confidence interval [CI] 2.3–25.2) in the risk of resulting in a stroke in comparison with other seasonal infections.^11^ Mechanisms of these strokes are explained by thrombo‐embolisms secondary to “cytokinic storm,” leading to vascular endothelial damage.^12^ It is still unknown if pathophysiology in strokes seen in children could be comparable to these seen in adults. Neurologic affections in SARS‐CoV2 infection in children are described in two literature reviews.^7^, ^13^ More than a half cases were associated with MIS‐C. The main two neurological manifestations described are headaches and acute encephalopathy.^14^ La Roviere et al. published a series of 12 cases of cerebrovascular disease in pediatric SARS‐CoV2 patients, but majority of them underlying risk factors.^15^ A systematic review of literature published in 2021, including 21 articles and 3707 patients found only 42 children with neurologic complications.^16^ All of them had a favorable short‐term prognosis. Plus, in an article published in 2022, an increased of seizures is described with the Omicron variant compared to the Delta variants.^17^

Stroke is a rare pathology in children. Most of them are seen in neonatal period. In older children, most of the causes are caused by infections, with vasculitis. Infectious agents known to be responsible for focal arteriopathy are Varicella‐zoster virus, herpes viruses, HIV, parvovirus B19, influenza A, enteroviruses, and *Mycoplasmia pneumoniae*. The delay between initial infection and neurologic symptoms can be long,^18^ sometimes around 6 months. The clinical presentation is various. In this case, neurologic symptoms appeared first, and COVID‐19 was diagnosed in PCR carried out in a systematic manner, with no symptom of tract respiratory infection.

Focal cerebral arteriopathy is described in children, but its management is rarely described.^19^ Pathophysiology of this clinical entity is still not understood.^20^ No guidelines exist concerning management of focal cerebral arteriopathy in children. A study published in 2017^21^ tried to compare combined corticosteroids and antithrombotic treatment versus antithrombotic treatment alone. No difference were made concerning the complete resolution of stenosis at 6 months after the initial episode (*p* = 0.197). “PASTA” study, a multicentric international study, is in progress to evaluate efficacy of corticosteroids in FCA in children. We decided to treat our patient with only acetylsalicylic acid and corticosteroids. Acetylsalicylic acid will be prescribed at least 18 months. We planned to control cerebral MRI with MRA at 6 months then at 12 and 24 months, if there is no intercurrent episode. The patient will be followed by neurologist, and regular evaluation of cognitive development will be made.

## 6 PATIENT PERSPECTIVE

This case report lead us to think about vaccination of SARS‐CoV2 in this age group. It is known that children affected by SARS‐Cov2 have mostly not severe infection. But last, with the new variants, neurological injuries are described, and it can be an argument to generalize vaccination in this target population.

## AUTHOR CONTRIBUTIONS

**Hortense Petat**: Conceptualization; investigation; methodology; supervision; validation; writing – original draft. **Adnan Hassani**: Investigation; writing – review and editing. **Ivana Dabaj**: Investigation; methodology; writing – review and editing. **Lucile Tzaroukian**: Investigation; writing – review and editing. **Barbara Goujard**: Investigation; writing – review and editing. **Isabelle Michelet**: Investigation; writing – review and editing. **Rebecca More**: Investigation; writing – review and editing.

## CONFLICT OF INTEREST

The authors declare no conflict of interest

## TRANSPARENCY STATEMENT

The lead author Hortense Petat affirms that this manuscript is an honest, accurate, and transparent account of the study being reported; that no important aspects of the study have been omitted; and that any discrepancies from the study as planned (and, if relevant, registered) have been explained.

## Data Availability

Research data are not shared.
